# Genetic Diversity within *Schistosoma haematobium*: DNA Barcoding Reveals Two Distinct Groups

**DOI:** 10.1371/journal.pntd.0001882

**Published:** 2012-10-25

**Authors:** Bonnie L. Webster, Aiden M. Emery, Joanne P. Webster, Anouk Gouvras, Amadou Garba, Oumar Diaw, Mohmoudane M. Seye, Louis Albert Tchuem Tchuente, Christopher Simoonga, Joseph Mwanga, Charles Lange, Curtis Kariuki, Khalfan A. Mohammed, J. Russell Stothard, David Rollinson

**Affiliations:** 1 Wolfson Wellcome Biomedical Laboratories, Department of Zoology, Natural History Museum, London, United Kingdom; 2 Department of Infectious Disease Epidemiology, Faculty of Medicine, Imperial College, St Mary's Campus, London, United Kingdom; 3 Réseau International Schistosomoses, Environnement, Aménagement et Lutte (RISEAL-Niger), Niamey, Niger; 4 Institut Sénégalais de Recherches Agricoles (ISRA), Bel Air, Dakar, Senegal; 5 Laboratoire de Parasitologie et Ecologie, Université de Yaoundé I, Yaoundé, Cameroon; 6 Center for Schistosomiasis and Parasitology, Yaoundé, Cameroon; 7 University of Zambia, Lusaka, Zambia; 8 National Institute for Medical Research, Mwanza, Tanzania; 9 Department of Invertebrate Zoology, National Museums of Kenya, Nairobi, Kenya; 10 Helminth Control Laboratory Unguja, Ministry of Health and Social Welfare, Zanzibar, Tanzania; 11 Disease Control Strategy Group, Liverpool School of Tropical Medicine, Liverpool, United Kingdom; University of Melbourne, Australia

## Abstract

**Background:**

Schistosomiasis in one of the most prevalent parasitic diseases, affecting millions of people and animals in developing countries. Amongst the human-infective species *S. haematobium* is one of the most widespread causing urogenital schistosomiasis, a major human health problem across Africa, however in terms of research this human pathogen has been severely neglected.

**Methodology/Principal Findings:**

To elucidate the genetic diversity of *Schistosoma haematobium*, a DNA ‘barcoding’ study was performed on parasite material collected from 41 localities representing 18 countries across Africa and the Indian Ocean Islands. Surprisingly low sequence variation was found within the mitochondrial cytochrome oxidase subunit I (*cox*1) and the NADH-dehydrogenase subunit 1 s*nad*1). The 61 haplotypes found within 1978 individual samples split into two distinct groups; one (Group 1) that is predominately made up of parasites from the African mainland and the other (Group 2) that is made up of samples exclusively from the Indian Ocean Islands and the neighbouring African coastal regions. Within Group 1 there was a dominance of one particular haplotype (H1) representing 1574 (80%) of the samples analyzed. Population genetic diversity increased in samples collected from the East African coastal regions and the data suggest that there has been movement of parasites between these areas and the Indian Ocean Islands.

**Conclusions/Significance:**

The high occurrence of the haplotype (H1) suggests that at some point in the recent evolutionary history of *S. haematobium* in Africa the population may have passed through a genetic ‘bottleneck’ followed by a population expansion. This study provides novel and extremely interesting insights into the population genetics of *S. haematobium* on a large geographic scale, which may have consequence for control and monitoring of urogenital schistosomiasis.

## Author Summary

Schistosomiasis is a disease caused by parasitic blood flukes of the genus *Schistosoma*. Species that infect humans are prevalent in developing countries, having a major impact on public health and well-being as well as an impediment to socioeconomic development. More people are infected with *Schistosoma haematobium* than with all the other schistosome species combined, however mainly due to the inability to maintain *S. haematobium* in the laboratory system empirical studies on this parasite are minimal. The genetic variation of this *Schistosoma* species on a wide geographical scale has never been investigated. In this study, we have used a DNA ?barcoding? approach to document the genetic variation and population structure of *S. haematobium* sampled from 18 countries across Africa and the Indian ocean Islands. The study revealed a distinct genetic separation of *S. haematobium* from the Indian Ocean Islands and the closely neighbouring coastal regions from *S. haematobium* found throughout the African mainland, the latter of which exhibited extremely low levels of mitochondrial diversity within and between populations of parasites sampled. The data from this study provides a novel insight into the population genetics of *S. haematobium* and will have an impact on future research strategies.

## Introduction

Schistosomiasis remains one of the world's greatest neglected tropical diseases (NTD). *Schistosoma haematobium* is one of the most widespread species of *Schistosoma* and causes urogenital schistosomiasis in humans. More people are infected with *S. haematobium* than with all the other schistosome species combined. Of the >110 million cases of *S. haematobium* infection in sub-Saharan Africa, 70 million are associated with hematuria, 18 million with bladder wall pathology, and 10 million with hydronephrosis leading to severe kidney disease [1]–[2] and even bladder cancer [3]. Despite the enormous numbers of people infected with *S. haematobium* and the pathogenesis of the parasite's infection, empirical studies on *S. haematobium* are minimal, compared to those of *S. mansoni* and *S. japonicum*, due, at least in part, to the inherent logistical difficulties of maintaining *S. haematobium* within the laboratory system [4]. However, as the whole-genome sequence of *S. haematobium* has recently been published [5], further research into this most neglected of the NTDs, at least at the genomic level, may now be facilitated.

*S. haematobium* has a large geographical distribution being found throughout Africa, parts of the Middle East, Madagascar and the Indian Ocean Islands and is transmitted by various intermediate snail hosts within the genus *Bulinus* [6]. As yet, the diversity of *S. haematobium* has been the subject of very few molecular studies [7]–[8], although one earlier study using enzyme analyses by isoelectric focusing in polyacrylamide gels to study 22 laboratory bred isolates of *S. haematobium* showed some regional variation and suggested mixing of parasite strains due to human population movements [9]. It remains imperative, however, that more investigations are conducted to elucidate the extent of genetic variation across the range of this parasite if we are to realistically understand its potential evolution, transmission and perhaps ultimate control.

Praziquantel (PZQ) remains the drug of choice for treatment of schistosomiasis and for the control of morbidity. It has a good safety and therapeutic record and is easy to administer (single oral dose), generally improving the health and well-being of schistosome-infected people. National control programmes in several sub-Saharan countries aim to alleviate the burden of schistosomiasis in highly endemic areas through large-scale administration of PZQ [10]–[13] and are likely to place strong and novel selective pressures on the parasites, which may be predicted to impact their population structure and genetics [14]–[16], [8].

In recent years, developments in molecular tools, and in particular advances in DNA sequencing, have allowed greater exploration and recording of the genetic diversity of schistosome species and their hosts thereof (e.g. [17]–[18], [16]). Sequence variation in the mitochondrial cytochrome oxidase sub-unit I (*cox*1) gene is commonly compared between sampled specimens to identify evolutionary differences, as well as, similarities. Such studies have benefited from knowledge of the complete mitochondrial genome of *S. haematobium*, as well as other schistosomes [19]–[21], thereby enhancing population-focused studies [22], [17], [4]. DNA ‘barcoding’ of a large number of populations of *S. mansoni* to date has revealed extensive population diversity with geographical structuring existing between populations [23]–[24]. A focused study on primarily laboratory passaged *S. haematobium* worms from Zanzibar also revealed substantial genetic diversity with the worms splitting into two distinct phylogenetic groups [25]. Taking a similar DNA ‘barcoding’ approach, the aim of this study was to document the genetic variation of *S. haematobium* from several areas geographically spread across Africa using historical collections of laboratory isolates but also mainly large collections of individual schistosome miracidia and cercariae sampled directly from their natural hosts, thereby avoiding the ethical and biological biases inherent within analyses of laboratory-passaged adult worms [18]. It was expected that the data would reveal any geographical structuring of the parasite populations and the extent of the genetic diversity within and between populations across Africa. Also, due to the wide geographic spread and extensive sampling of miracidia, primarily from infected school-aged children it was predicted that the same or more genetic diversity would be found within and between populations as that found in the study on Zanzibar [25]. By so doing the origin, evolution and spread of *S. haematobium* on the African mainland and the Indian Ocean Islands could be further elucidated.

### Ethical statement

Ethical approval was obtained from Imperial College Research Ethics Committee (ICREC), Imperial College London in the UK, in combination with the ongoing Schistosomiasis Control Initiative (SCI) activities. In Senegal, ethical approval was obtained from the ethical committees of the Ministry of Health Dakar, Senegal. In Niger, ethical clearance was obtained from the Niger National Ethical Committee. In Cameroon, ethical approval was obtained from the Commité National d'Ethiqué, Cameroon. In Kenya, ethical approval was obtained from the Ethical Review Board of National Museums of Kenya/Kenya Medical Research Institute. In Tanzania, ethical approval was obtained from the Ethical Review Board of National Institute of Medical Research (NIMR). In Zambia, ethical clearance was obtained from the University of Zambia ethics committee.

Before conducting the study, the MoH-approved plan of action had been presented and adopted by regional and local administrative and health authorities. Meetings were held in each village to inform the village leader, heads of the families, local health authority, teachers, parents and children about the study, its purpose and to invite them to voluntarily participate. According to common practice and with approval from the Imperial College Research Ethics Committee (ICREC), due to low levels of literacy all village leaders, teachers, parents and study participants gave oral consent for the studies to take place. Informed consent for the urine examinations was obtained from each study participant and their parents or guardians. Oral consent for each participant was documented by inscription at school committees comprising of parents, teachers and community leaders. All the data were analyzed anonymously and all schistosomiasis positive participants were treated with PZQ (40 mg/kg). In schools or classes where the percentage of infections were more than 50%, mass treatment of all children was carried out at the end of the study.

The historical material stored in SCAN has been derived from UK laboratory passage of original specimens collected in collaboration with local authorities abiding by the ethical standards and collection requirements of the day and all specimens were maintained and analyzed anonymously.

Laboratory animal use was within a designated facility at the NHM regulated under the terms of the UK Animals (Scientific Procedures) Act, 1986, complying with all requirements therein, including an internal ethical review process at the NHM and regular independent Home Office inspection. Work was carried out under the Home Office project license numbers 70/4687 (-2003), 70/5935 (2003–2008), 70/6834 (2008-).

## Materials and Methods

### Sample collections

As part of a European Commission Specific Research Project (CONTRAST) ‘A multidisciplinary alliance to optimize schistosomiasis control and transmission surveillance in sub-Saharan Africa’, parasitological surveys were conducted at 13 localities in a total of seven countries across Africa between 2007–2010 (Table S1). Eggs were sampled directly from urine samples, either individually or pooled, of infected children. Eggs were concentrated from each infected urine sample by sedimentation or filtration, then rinsed in saline before transfer into a clean Petri dish containing mineral water and exposed to light to facilitate hatching of miracidia. Using a binocular microscope, individual miracidia were then captured in 2–5 µl of mineral water, pippetted onto Whatman FTA cards and allowed to dry for 1 hour [18].

Individual cercariae from naturally infected snails were also sampled from a few localities (Table S1). Snails collected from known transmission sites were placed individually or pooled into pots of fresh mineral water and exposed to light to stimulate cercarial shedding. On inspection, using a binocular microscope, visually identified schistosome cercariae were captured in 2–5 µl of mineral water, pipetted onto Whatman FTA cards and allowed to dry for 1 hour.

Laboratory passaged adult worms from nine additional localities held in the Natural History Museum, London (NHM) liquid nitrogen schistosome repository, Schistosome Collections at the Natural History Museum (SCAN), were also utilised for molecular analysis (Table S1). A feature of the biology of schistosomes relevant to the use of laboratory passaged samples for molecular analysis is that there will be a high level of selection or genetic bottlenecks imposed on the population during the passaging process and so these samples cannot realistically be treated as individual samples nor used to represent the true within-locality variation from these particular isolates [18]. Nevertheless, we chose to include these additional archived adult worm samples to increase the geographic range and scope of the current study. Individual worms sampled from the same laboratory passaged isolate/NHM number were not treated as individuals, but the different haplotypes found were used as representative data from those localities.

### DNA extraction and amplification

#### Whatman FTA stored samples

For genomic DNA (gDNA) extraction, a 2.0 mm disc containing the sample was removed from the card using a Harris-Micro-Punch (VWR, UK) and incubated for 5 mins in 200 µl of FTA purification reagent (Whatman plc. Maidstone, Kent). The FTA purification reagent was removed and the disc was incubated for a further 5 mins in 200 µl of fresh FTA purification reagent. This process was repeated for a total of 3 washes and was followed by 2×5 mins incubations in 200 µl of TE buffer. Samples were air dried at 56°C for 10–30 mins and the disc was visually checked to make sure it was dry before being used directly in PCR reactions.

### Worms held in liquid Nitrogen

Adult worms were thawed on ice and total gDNA was extracted from individual males and females Table S1, using the DNeasy Blood and Tissue Kit (Qiagen Ltd, Crawley, UK) and eluted in 100 µl of buffer giving a concentration of 3.6–31.5 ng/µl of gDNA from each worm, 2 µl of which was used for PCR.

### DNA amplification and sequencing

#### PCR of the partial region of the mitochondrial (mt) cytochrome oxidase (*cox*1) gene

For each individual worm, miracidium or cercaria, 956 bp of the mt *cox*1 gene was amplified in separate 25 µl PCR reactions using illustraTM puReTaq Ready-To-Go PCR Beads (GE Healthcare, UK) and 10 pmol of each primer (Forward primer: COX1_Schisto_5′; Reverse primer; COX1_Schist_3′ [26]. Thermal cycling was performed in a Perkin Elmer 9600 Thermal Cycler and the PCR conditions used were: 5 mins denaturing at 95°C: 40 cycles of 30 sec at 95°C, 30 sec at 40°C, 2 mins at 72°C; followed by final extension period of 7 mins at 72°C. Four µls of each amplicon was visualised on a 0.8% gel-red agarose gel and positive PCR's were purified using the QIAquick PCR purification Kit (Qiagen Ltd, UK) and then sequenced on an 3730XL 96 capillary automated sequencer (Applied Biosystems, UK) in both directions using 1.6 pmol dilutions of the original PCR primers and internal sequencing primers *S.haem*_*cox1*_F + *S. haem*_*cox1*_R [25] and an Applied Biosystems Big Dye Kit (V1.1).

#### PCR of the partial region of the mitochondrial (mt) NADH-dehydrogenase subunit 1 (*nad*1) gene

To provide data from a different mt gene, the partial NADH-dehydrogenase subunit 1 (*nad*1) (756 bp) was amplified and sequenced as above using DNA available from several of the laboratory passaged samples (Table S1). The primers used were *S.haem*_*nad1_*F and *S.haem*_*nad1*_R [25].

### Data analysis

#### mt DNA sequence analysis

All sequences for the different (*cox*1 and *nad*1) datasets were assembled and manually edited using Sequencher V4.6 (http://genecodes.com) to remove any ambiguities between forward and reverse strands. For each sample consensus sequences were aligned in Sequencher and polymorphic positions observed between individuals were checked and confirmed by visualisation of the original sequence chromatograms. The identity (species and gene) of the sequence was also confirmed using the Basic Local Alignment Search Tool (NCBI-Blast). Within each individual locality (see locality codes Table S1) consensus sequences from each individual sample were grouped and aligned in MacClade 4.05 and then collapsed together using Collapse V1.2 (http://darwin.uvigo.es/software/collapse.html) to identify individual samples with identical sequences. Each group of identical sequences and also any unique sequences became unique haplotypes for that site and consensus sequences were created and given a unique haplotype identifier code, consisting of the site code and a letter representing the different haplotypes within each locality. The numbers of individuals that presented each haplotype in each locality were also recorded.

#### Published mitochondrial data from other isolates of *S. haematobium*

Included in our analysis was *S. haematobium cox*1 and *nad1* data available on the NCBI database (http://www.ncbi.nlm.nih.gov), which was downloaded and incorporated into the datasets from our collections (Table S1). The Zanzibar *S. haematobium* data from Webster *et al.* [25], was also included in the analysis.

#### Haplotype analysis

The complete datasets (sequences of the unique haplotypes from each locality found in this study and the published data) were aligned in MacClade 4.05, and then collapsed (Collapse V1.2 (http://darwin.uvigo.es/software/collapse.html) to identify any identical haplotypes from different localities.

Haplotypes that were identical to the most common haplotype found across Africa (H1, Table S1) were noted. The sequence of this main (*cox*1 or *nad*1) haplotype (H1) was used in the analyses to represent all the individuals that it corresponded to, unless stated otherwise. Haplotype sequences were submitted to EMBL/Genbank (Table S1).

To estimate genealogical relationships between haplotypes, the individual haplotype sequences were aligned in MacClade V4.05 and then a minimum spanning network was created in the programme TCS (http://darwin.uvigo.es/software/tcs.html).

#### *cox*1 phylogenetic analysis

All the *cox*1 haplotype sequences were aligned in MacClade V4.05 and exported into Mega V5 [27]. Evolutionary relationships between the haplotypes were inferred using the Neighbour-Joining, Maximum Parsimony and Minimum Evolution methods using the Kimura's 2-parameter model (K2P) for pair-wise distance calculations. Analyses were subjected to 1000 bootstraps to test the reliability of branches of the trees. The topologies were rooted by the sister species *Schistosoma bovis* for which sequence data was obtained, using the methods described above, from a Senegal isolate that had been preserved in the NHM liquid nitrogen schistosome repository, SCAN. To test the topology of the tree further a Maximum Likelihood (ML) analysis with 500 replicates was also instigated in Mega V5 using the best fit ML model (HKY+G) which was calculated in Mega V5 and also by using the Akaike criterion in jModeltest V0.1.1 [28].

The net nucleotide divergence (*D_a_*) between the two main groups found in this analysis was calculated with the Juke-Cantor correction model in DnaSP V5.

#### *cox*1 population genetic analysis

To analyze the population *cox*1 diversity the sequences from each individual sample were aligned in MacClade 4.05 and exported into DnaSP V5 [29]. These were then used to calculate haplotype diversity (*h*) and nucleotide diversity (Π), the latter of which was calculated with Juke-Cantor corrections, which was the most complex substitution model available. Overall diversity was measured together with the diversity within different geographic regions and also each locality (Tables 1 & 2). All individual miracidia and cercariae were treated as independent samples and their individual sequences were incorporated into the analysis. In contrast, for samples obtained from laboratory passage, and therefore probably highly clonal as discussed above, only individual worms with different sequences were used in the analyses. No laboratory passaged samples were included in the within-locality analysis (Table 2).

**Table 1:** Overall and regional *cox*1 diversity.

Region	*n*	*u*	*h*	∏
**All**	1978	61	0.358±0.014	0.00435
**Mainland Africa**	1682	24	0.139±0.011	0.00068
**Indian Islands** ***	296	43	0.926±0.009	0.01177
**East Africa**	197	21	0.829±0.020	0.01129
Kenya*	20	1	0	0
Tanzania	23	3	0.170±0.102	0.004
Malawi*	5	2	0.400±0.237	0.00042
Zambia	71	2	0.028±0.027	0.00006
Coastal Kenya	78	15	0.874±0.021	0.01216
**Egypt** *	2	2	1.0±0.5	0.00113
**Sudan** *	1	1	0	0
**Central West Africa**	208	10	0.121±0.031	0.00013
Cameroon	207	10	0.121±0.031	0.00013
Nigeria*	1	1	0	0
**Far West Africa**	1353	10	0.026±0.006	0.00003
Niger	136	2	0.029±0.020	0.00003
Senegal	1211	7	0.023±0.006	0.00002
Mali*	3	2	0.667±0.314	0.00070
Gambia*	1	1	0	0
Liberia*	1	1	0	0
Guinea Bissau*	1	1	0	0
**South Africa** *	1	1	0	0
**Mauritius** *	1	1	0	0
**Madagascar** *	1	1	0	0
**Zanzibar** **	214	27	0.893±0.013	0.01137

*n* = number of samples sequenced; *u* = number of unique haplotypes found within the region; *h* = haplotype diversity ± standard deviation; **∏** = nucleotide diversity.

* samples form laboratory passaged or pooled larval stages. For these samples individual worms were not treated as individual samples but instead each different haplotype found was treated as an individual sample.

** For Zanzibar the whole data set of Webster et al. [25], was used and due to the high number of worms sampled and high diversity found, individual worms were treated as individual samples. This was also used in the analysis of the diversity overall and in the Indian Island region.

*** This included samples from Zanzibar, Madagascar, Mauritius and Mafia.

**Table 2:** Within locality *cox*1 diversity.

Locality*	*n*	*u*	*h*	∏
**Senegal**				
Nder, SE3	397	2	0.005±0.005	0.00001
Temeye, SE4	120	1	0	0
Podor, SE5	104	1	0	0
Tambacounda, SE6	288	2	0.014±0.010	0.00001
Kolda, SE7	10	2	0.356±0.159	0.00037
Barkedji SE8	288	3	0.055±0.019	0.00006
**Niger**				
Libore, NI1	96	2	0.041±0.028	0.00004
Falmado, NI2	40	1	0	0
**Cameroon**				
Bessoum, CA1	193	10	0.130±0.033	0.00014
Okuro, CA2	10	1	0	0
**Kenya**				
Taveta, KE1	20	1	0	0
Coastal Kenya				
Rekeke, CK1	39	8	0.695±0.066	0.01207
Kinango. CK2	24	4	0.576±0.097	0.00532
Nimbodze, CK3	15	4	0.752±0.056	0.00312
**Tanzania**				
Mwanza, TA1	21	2	0.095±0.084	0.00243
Zanzibar, Zan	214	27	0.893±0.013	0.01200
**Zambia**				
Katunga. ZA2	20	1	0	0
Kafue, ZA3	18	1	0	0
Lisiko, ZA4	32	1	0	0

*n* = number of samples sequenced; *u* = number of unique haplotypes found within the region; *h* = haplotype diversity ± standard deviation; **∏** = nucleotide diversity.

* Only localities where miracidial populations were collected and the data from Zanzibar [25] were included.

The Genbank Accession numbers for the *cox*1 data are JQ397330–JQ397399 and for the *nad*1 data are JQ595387–595404 (see Table S1).

#### Tests of selection

In DnaSP V5 the McDonald_Kreitman test for selection and Tajima's test of neutrality were conducted on our *cox*1 data to investigate if there was significant selection occurring.

#### *nad*1 phylogentic analysis

*Nad*1 data was only available from a subset of the samples from 18 different localities and only from laboratory passaged worms or previously published data. Therefore, these data were only analyzed at a basic phylogenetic level and not the population level. The *nad1* haplotypes were aligned in MacClade V4.05 and exported into Mega V5 [25]. Evolutionary relationships between the haplotypes were inferred using the Neighbour-Joining method and 1000 bootstraps to test the reliability of branches of the trees. The topologies were rooted by the sister species *S. bovis* as described for the *cox*1 data.

### Analysis of a nuclear marker

To compare variation found within the mtDNA with nuclear DNA, the complete ITS (1+2) rDNA (927 bp) was amplified from a single male and a single female worm from all localities that had representative adult worms available (Table S1). This marker was amplified and sequenced with the forward and reverse primers ITS1 + ITS2 [30] using the PCR and sequencing conditions as used for *cox*1. In the analysis, published *S. haematobium* ITS data were also included from Senegal (FJ588861), Mali (Z21716) and Zanzibar (GU257398). Sequences were aligned and any nucleotide differences recorded.

## Results

### Sequence data

There were no differences between the ITS1+2 sequences from any of the samples. This nuclear marker therefore proved uninformative as a population genetic marker for *S. haematobium*.

In total 1978 *cox*1 sequences were analyzed: 46 from cercariae, 241 from laboratory passaged adult worms (35 published haplotype sequences from previous studies of which 27 were from 214 individual worms isolated from Zanzibar [25] and 1869 from miracidia collected directly from their human hosts. The *cox*1 region (956 bp) analyzed contained 70 variable sites (53 parsimony informative), and the diversity found within the *S. haematobium* populations sampled was low with the sequences resolving into just 61 unique haplotypes. The percentage occurrence of each haplotype varied but there was a main common haplotype (H1) that was found across most of the localities and appeared frequently on mainland Africa, representing 1574 (80%) out of the 1978 overall sequences analyzed.

**Figure 1:**
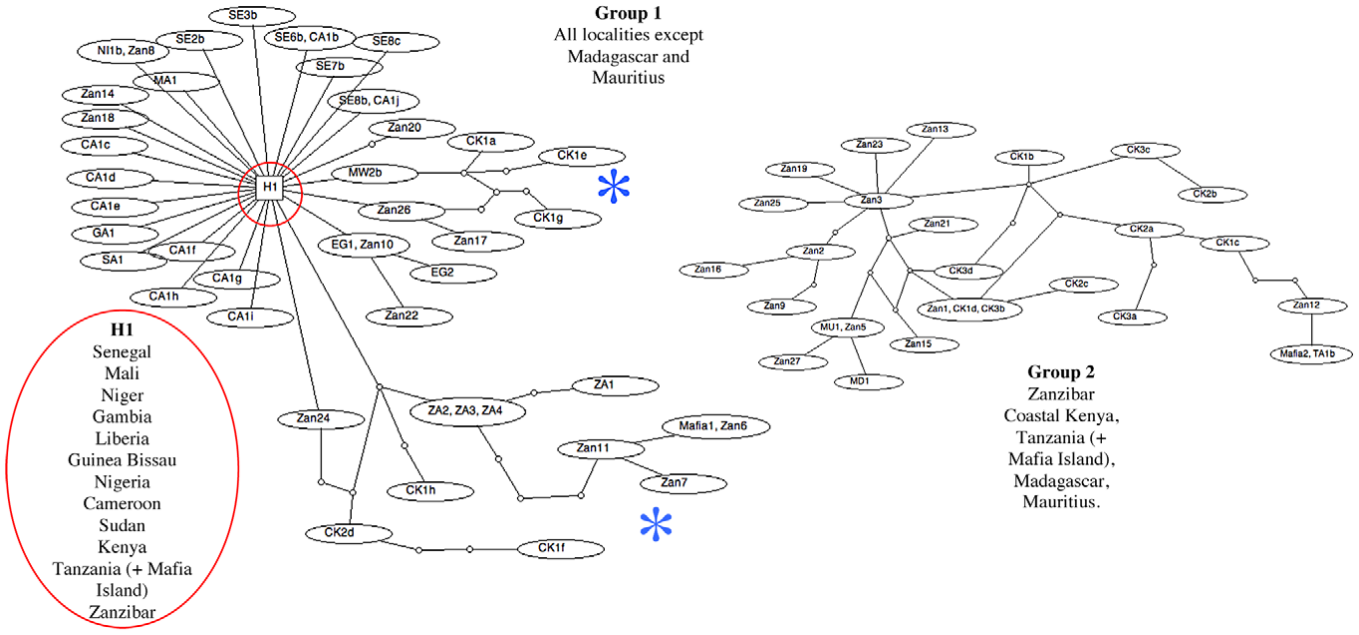
Minimum spanning TCS networks incorporating all the *cox*1 haplotypes analyzed in this study. Each line between haplotypes represents a single bp change and small circles between lines represent unsampled or extinct haplotypes. The network forms 2 distinct groups of haplotypes that cannot be linked. Group 1 is a simple network containing the main land African samples and a few of the Indian Ocean Island samples. The majority of the samples are closely clustered around the main haplotype (H1) by separate single links representing a single polymorphic position. There are 2 longer branches, “blue star”, leading off from the main cluster to form networks between Zanzibar, Coastal Kenya, Tanzania (+Mafia) and Zambian samples. H1 represents samples from 29 out of the 43 separate localities and represents 1574 out of the 1978 sequences analyzed (see figure 2 for the list of haplotype codes that represent H1). Group 2 forms a more complicated network containing the majority of the samples from the Indian Ocean Islands and samples from the closely located areas of Coastal Kenya and Tanzania. Identical haplotypes are grouped in the same oval.

The minimum spanning TCS network (Figure 1) clearly shows the dominance of H1 and splits the haplotypes into two groups, one that is made up of 28 haplotypes from Zanzibar, Coastal Kenya, Tanzania, Mafia Island, Madagascar and Mauritius and one that is made up of 46 haplotypes from all localities sampled excluding Madagascar and Mauritius. The two groups could not be linked due to too many missing steps: the network for Group 1 is more basic with H1 being a dominant central point from which, many of the other haplotypes branch off by 1 single step (1 bp change). This closely linked network, linked by single bp changes, is made of haplotypes predominantly found in mainland Africa (except Zambia) and a few haplotypes from Zanzibar. The majority of these single mutations do not form links with other haplotypes suggesting that they are random mutations that come and go but do not persist within the populations. Exceptions to this are the Egyptian haplotypes, which again branch off from the main haplotype H1 by one mutation and forms two haplotypes separated by a single bp change. The other more significant exceptions are longer branches forming more complicated networks with haplotypes from Zanzibar and its neighbouring regions Coastal Kenya and Mafia Island and also samples from Zambia. Both branches are again closely linked to H1 by single mutations with one branch also forming links with a Malawi haplotype and the other incorporates the haplotypes from Zambia. Group 2 forms a more complicated network between haplotypes and is more exclusive containing haplotypes from only the Indian Ocean Islands and the neighbouring East African regions of coastal Kenya and Tanzania.

### Phylogenetic structuring

**Figure 2:**
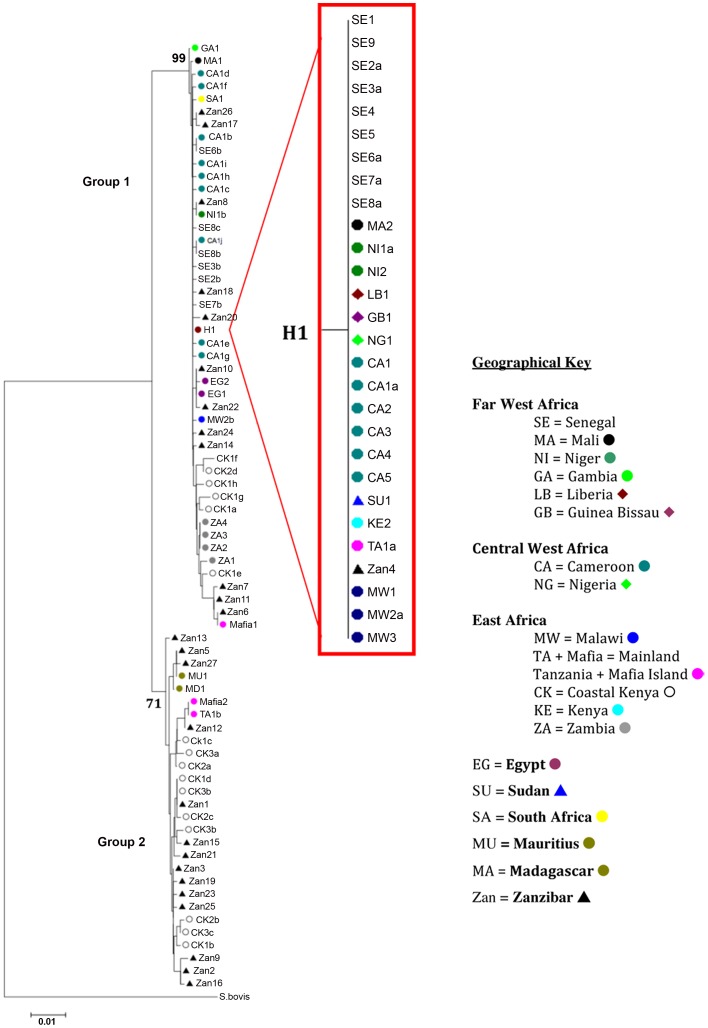
Neighbour-joining *cox*1 tree topology. Nodal supports for the 2 groups are marked and details of the samples representing H1, “red dot”, are shown in the sub tree. Each terminal branch is labelled with the individual haplotype codes as detailed in Table S1.

As with the TCS analysis the same general splitting of the haplotypes was found with all phylogenetic methods separating the 61 haplotypes into two distinct well-supported groups (Figure 2) with H1 being central to the majority of the mainland African haplotypes. The details of all the samples that represent H1 can be seen in the sub tree in Figure 2. The tree topologies show the separation of samples from Coastal Kenya, Zanzibar, Mafia Island and Zambia from the main cluster in Group 1 and also the clear separation of Group 2 containing samples from the Indian Ocean Islands and its neighbouring African coastal regions.

The net divergence between the groups (0.02145±0.00102) shows a relatively short time between the genetic separation of these two *S. haematobium* groups compared to that of the much larger divergence from their sister taxa *S. bovi*s (Group 1: 0.11644±0.01978, Group 2: 0.10932±0.02365)

### Population genetics

Measures of overall haplotype and nucleotide diversity, together with within region and locality diversity, are presented in Tables 1 and 2. Sample numbers will affect the diversity especially when very low levels of samples are used, however the data do show a clear difference in diversity between those localities where large numbers of individual larvae were sampled. The number of unique haplotypes found in the western regions of Africa compared to the east is extremely small even though sampling was biased towards the west and the diversity seen in the east comes mainly from the coastal Kenyan samples. There is also a vast contrast, with an extremely high diversity found within the populations sampled from the Indian Ocean Islands and the neighbouring coastal regions compared to the rest of Africa, where one dominant haplotype appears to persist throughout the mainland. We also tested for strong selection in our data and found no deviation from neutral expectations (*p* values were all > than 0.05).

### *nad1 S. haematobium* data

**Figure 3:**
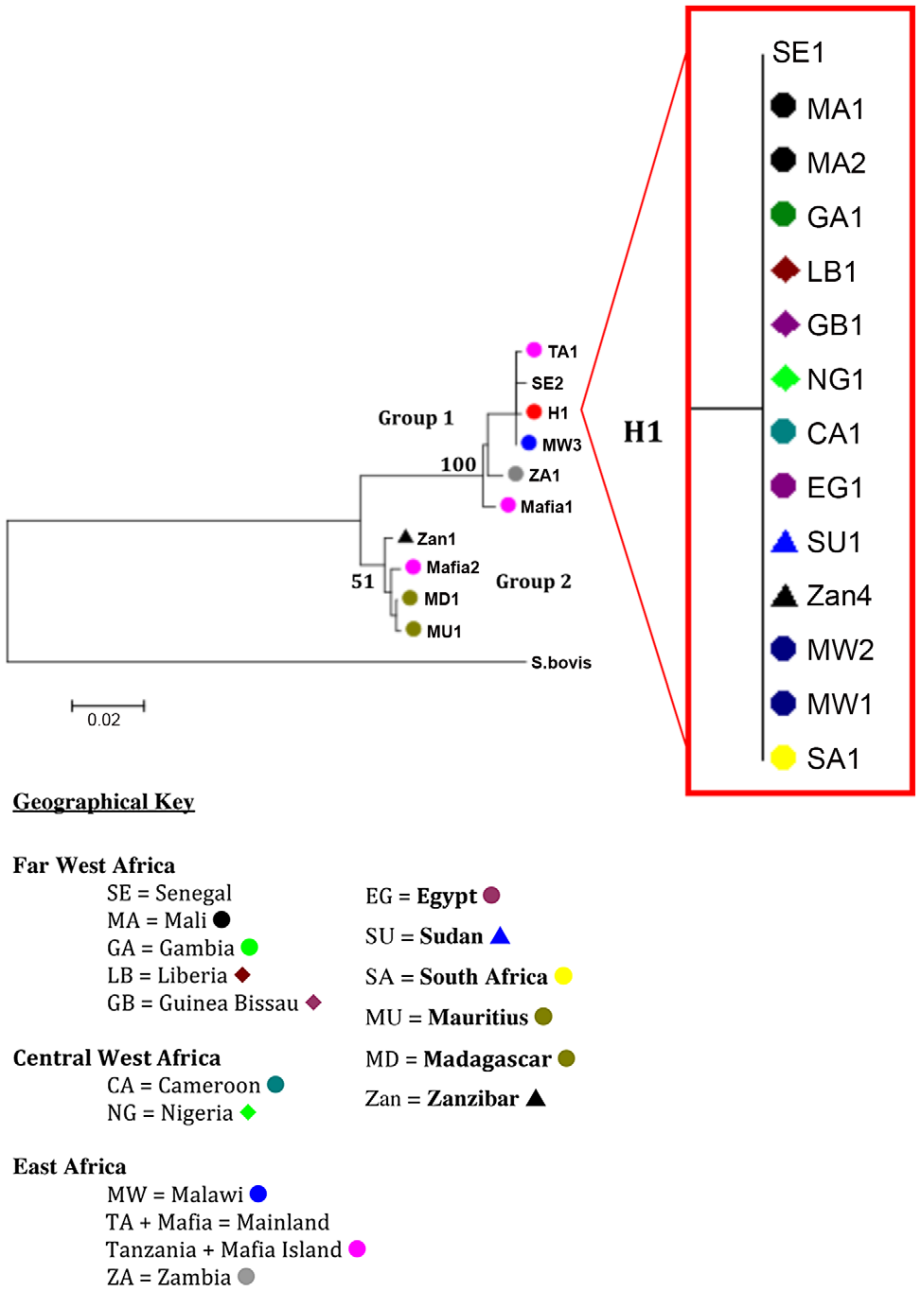
Neighbour-joining *nad*1 tree topology supporting the topology of the *cox*1 tree. Nodal supports for the 2 groups are marked and details of the samples representing H1, “red dot”, are shown in the sub tree. Each terminal branch is labelled with the individual haplotype codes as detailed in Table S1.

The *nad*1 haplotypes sequenced from several of the samples (Table S1) also supported the findings from the *cox*1 data (Figure 3). The haplotypes again split into two distinct groups; 1) dominated by a central haplotype found throughout mainland Africa and Zanzibar with a few closely linked haplotypes forming short branches and a longer branch to the Zambian haplotype and 2) containing haplotypes from Zanzibar, Madagascar and Mauritius.

## Discussion

DNA ‘barcoding’ approaches are now commonly used to provide insights into population structure and diversity within species including schistosomes. Studies on *S. mansoni* have shown high levels of mtDNA diversity within and between populations from endemic areas with haplotypes segregating by geography [23]–[24].

This study is the first time that DNA *cox1* ‘barcoding’ has been used to elucidate the genetic diversity of *S. haematobium* populations across Africa and also from several of the Indian Ocean Islands (Zanzibar, Madagascar, Mauritius, Mafia). Ninety seven percent of the data generated came from larval stages sampled directly from their human hosts from across 20 localities, with the remainder of the data coming from historical collections based on laboratory-passaged worms. The study has revealed that the genetic diversity of *S. haematobium* across Africa is unexpectedly low. There were only 61 unique haplotypes found in the 1978 samples collected from 41 locations and 18 countries. The haplotypes split into two distinct groups; one that contains haplotypes predominately from mainland Africa with a few haplotypes from Zanzibar (Group 1) and the other that is made up of samples exclusively from the Indian Ocean islands and the neighbouring African coastal regions (Group 2). The net divergence between the two groups was considerable and was strongly supported by both the *cox*1 and the *nad*1 data. This is equivalent to the net divergence seen between some *S. mansoni* groups spread across Africa, separated by thousands of miles [24].

The lack of the diversity found within and between the *S. haematobium* samples can clearly be seen in the TCS network and phylogenetic analyses with a single haplotype (H1 from 1574 samples) being dominant across Africa with greater diversity found within the samples from the Indian Ocean Islands and the neighbouring African coastal regions. The nuclear ITS data showed no diversity from any sample proving that this can not be used as a population genetic marker for this parasite [24] although such a nuclear marker is vital for the detection of interactions with closely related species and for confirming species identity [31].

The longer branches stemming from the main H1 haplotype in Group 1 on the TCS analysis show that the populations from coastal Kenya, Zambia and Mafia are quite separated from the main haplotype group. The highest diversity was found in the *S. haematobium* populations from coastal Kenya and Zanzibar with complicated networks linking these haplotypes and several nodes not being represented by a haplotype. This suggests that haplotypes may have become extinct or that they have not been sampled indicating there may be more diversity still to be discovered in these areas. However, the basic network of single links around H1 suggests that further sampling in the other areas, reported on in this study, is unlikely to reveal further discrete groupings. With exceptions of haplotypes from the Indian Ocean Islands, Coastal Kenya and Zambia, the network clearly shows a lack of geographic structuring with the same *cox*1 haplotypes being found in Far West, Central West, East, South of Africa and also in Sudan, Egypt and Zanzibar.

The distribution of the haplotypes must reflect in part past movements of people. Group I and 2 parasites have been isolated from the same geographical regions and from the same host. For example, the haplotypes from Mwanza, Tanzania (TA1a + b), resolve into both network groups, one haplotype (TA1a) being identical to the H1 haplotype (Group 1) and one (TA1b) being identical to a haplotype found on Mafia Island and sitting at the end of a long branch within Group 2. This suggests that in this geographic region as well as the main dominant *S. haematobium* genotype there has also been transfer of Group 2 schistosomes from the Indian Ocean Islands and the neighbouring coastal regions probably associated with the movements of people [32]. Similarly, the positioning of the Mafia haplotypes (Mafia1 + 2) in the two groups shows the close affinity of this population with *S. haematobium* from Zanzibar. This is not unexpected due to infected children from Mafia having a travel history to the Zanzibar mainland and that urogenital schistosomiasis is suggested to be imported and not endemic on Mafia.

The higher diversity found in the coastal Kenyan populations (15 haplotypes), the close clustering with the Zanzibar haplotypes and the separation of these populations into the two groups also suggests a close association between parasites from coastal Kenya and Zanzibar. There is probably mixing and movements of the populations between coastal Kenya and Zanzibar and vice versa with the movements of people between these areas due to the trade routes between the Indian Ocean islands and the neighbouring East African coastal regions [32]. The positioning of the haplotypes from Madagascar and Mauritius in Group 2 further supports the uniqueness of the haplotypes from the Indian Ocean Islands and neighbouring East African coastal regions compared to those from mainland Africa. The recognition and distribution of Group 2 schistosomes suggests movement of *S. haematobium* populations from endemic adjacent regions such as Madagascar and the Arabian Peninsula. There is a history of prolific trade links between the Arabian Peninsula, India, the Indian Ocean Islands and the East coast of Africa aided by the Monsoon trade winds, which probably would have facilitated the movements of people and their parasites between these areas.

In consideration of general population genetic theories, the extremely low levels of genetic diversity between the *S. haematobium* populations separated by 1000's of miles across continental Africa compared to the high diversity found within the populations from the more isolated Indian Ocean Islands and their closely neighbouring African coastal regions is unexpected. It is particularly striking that the dominant haplotype H1 occurs across Africa with 1574 samples analyzed not showing a single nucleotide mutation in the mtDNA analyzed. The success of this haplotype might be attributed to a founder effect following a population ‘bottleneck’ with only a few individual parasites surviving and participating in a later population expansion. The lack of diversity found across Africa and the restriction of the Group 2 haplotypes to coastal regions of East Africa and the Indian Ocean Islands suggests that this may have happened relatively recently in terms of the evolutionary history of these parasites. Given the close phylogenetic relationship of the Indian/Asian and African *Schistosoma* species [33]–[34] it is possible that the lack of genetic diversity found within and between the *S. haematobium* populations across mainland Africa is attributed to a re-invasion by a small number of individuals of *S. haematobium* into Africa from a larger population in Asia across the Arabian peninsula, with a subsequent rapid spread and population expansion across Africa from East to West. The new small re-established population in Africa would be more sensitive to genetic drift and increased inbreeding resulting in low genetic variation. Due to the lack of fossil records it is extremely difficult to accurately define the evolutionary history and phylogeography of the *Schistosoma* genus [24], [34]–[35] however, data such as that reported here do provide new insights into how these parasites evolved and spread and c learly it will be of interest to barcode samples from the Arabian Peninsula.

One factor that could have influenced the divergence of our populations into the two groups relates to compatibility with intermediate snails hosts. Intermediate host use of *S. haematobium* is very specific and varies in different geographical locations [6]. However, based on current day snail distributions there is no obvious correlation between intermediate host use and the observed parasite diversity. *S. haematobium* in East Africa and the Indian Ocean Islands is mainly transmitted by *Bulinus africanus* and *B. forskalii* group species while elsewhere the same species groups can be involved but snails of the *B. truncatus tropicus* complex may also play an important role in transmission. However, the study by [36] did show that the intermediate snail host, *B. globosus*, separates into distinct West and East African clades on a molecular phylogenetic tree, possibly suggesting that the distribution of East African *B. globosus* could be a limiting factor in the spread of Group 2 type parasites. It is clear that more studies are needed to investigate the role of the different snail species and geographical populations of *Bulinus* in the transmission of Group 1 and Group 2 type parasites. As well as intermediate snail host compatibility it will be important to determine whether the different groups give rise to infections which result in different pathologies or which respond differently to treatments as genetic diversity has been noted to possibly have an effect on such characteristics [37].

Though the small regions of nuclear DNA analyzed in this study proved highly conserved, it is likely that there could be more diversity found in other regions of the nuclear genome, which may or may not correlate to that found in the mt DNA. A recent study [8], using a small number of microsatellite markers did find diversity in *S. haematobium* miracidial populations from Mali conflicting the data presented here, however only laboratory maintained material from Mali was analysed in the present study and so a direct comparison cannot be made. Due to the difficulties in directly sampling natural schistosome populations there exists a strong sampling bias within this study with the majority of the data obtained from large larval schistosome populations collected from 6 countries. The other countries are represented by far fewer or laboratory maintained samples and whilst providing useful genetic data it cannot be concluded that they are representative of the true genetic diversity in these countries. More samples need to be analyzed from more areas and with more genetic markers to further elucidate the genetic diversity of *S. haematobium* populations from all it's endemic areas. It would also be beneficial to analyze both nuclear and mt DNA simultaneously from the same individual sample in future population genetic studies and the recent publication of whole-genome sequence of an Egyptian isolate of *S. haematobium* [5] will facilitate the development of many more nuclear markers for population genetic analyses at the genomic level.

The genetic diversity of schistosome populations can be influenced by a variety of factors such as; host water-contact patterns, host immunity and susceptibility and moreover, mass chemotherapy has a great potential to promote selection. [15], [8]. The impact of the large-scale administration of PZQ, through national control programmes [11]–[12] on the genetic selection of both *S. mansoni* and *S. haematobium* is an area of high interest with respect to the development of drug resistance [38]–[40], [14]–[15], [41]. A high population diversity would be expected to provide a wide genetic base for selection to act upon possibly increasing the rate of resistance to treatment developing, ultimately resulting in a decline in diversity over time to a few, non susceptible genotypes [42]. The relatively low level of diversity within *S. haematobium* across most of mainland Africa, as defined by the current genetic markers, may indicate that these parasites may be less likely to change under drug pressure, however the high genetic diversity found on Zanzibar and the neighbouring African coastal region could offer a genetic base for the development of PZQ resistance and hence changes in parasite diversity in relation to chemotherapy needs to be monitored in these highly diverse areas.

This study has reported on some very unusual findings in relation to *S. haematobium* mtDNA population genetics. It is clear that further sampling in many areas will not dramatically increase the mtDNA diversity found but in areas such as Zambia, the East African coastal regions and the Indian Ocean islands where more diverse populations of *S. haematobium* have been found, further sampling would add to our understanding of the parasite population movements and diversity. It is also important that the population genetics of the *S. haematobium* is monitored further to link diversity with morbidity and to provide information on the response of parasite populations to drug treatment pressures. The mtDNA diversity described here, together with other molecular markers, will be of value to monitor the impact of control interventions on different *S. haematobium* genotypes and may assist in understanding the introduction or re-introduction of parasites associated with human population movements.

## Supporting Information

Table S1 **Sample and haplotype information (Supporting Table).**
(DOC)

## References

[1] King CH, Dickman K, Tisch DJ (2005) Reassessment of the cost of chronic helmintic infection: A meta-analysis of disability-related outcomes in endemic schistosomiasis. Lancet 365: 1561–1569. 15866310 10.1016/S0140-6736(05)66457-4

[2] van der Werf MJ, Bosompem KM, de Vlas SJ (2003) Schistosomiasis control in Ghana: Case management and means for diagnosis and treatment within the health system. Trans R Soc Trop Med Hyg 97: 146–152. 14584366 10.1016/s0035-9203(03)90102-7

[3] Shiff C, Veltri R, Naples J, Quartey J, Otchere J, et al (2006) Ultrasound verification of bladder damage is associated with known biomarkers of bladder cancer in adults chronically infected with *Schistosoma haematobium* in Ghana. Trans R Soc Trop Med Hyg 100: 847–854. 16443246 10.1016/j.trstmh.2005.10.010

[4] Rollinson D (2009) A wake up call for urinary schistosomiasis: reconciling research effort and public health importance. Parasitology 136: 1593–1610. 19627633 10.1017/S0031182009990552

[5] Young ND, Jex AR, Li B, Liu S, Yang L, et al (2012) Whole-genome sequence of *Schistosoma haematobium* . Nature Gen 44: 221–225. 10.1038/ng.1065 22246508

[6] Rollinson D, Stothard JR, Southgate VR (2001) Interactions between intermediate snail hosts of the genus *Bulinus* and schistosomes of the *Schistosoma haematobium* group. Parasitology 123: 245–260. 10.1017/s0031182001008046 11769287

[7] Brouwer KC, Ndhlovu PD, Wagatsuma Y, Munatsi A, Shiff CJ (2003) Urinary tract pathology attributed to *Schistosoma haematobium*: does parasite genetics play a role? Am J Trop Med Hyg 68: 456–462. 12875296

[8] Gower CM, Gabrielli AF. Sacko M, Dembelé R, Golan R, et al (2011) Population genetics of *Schistosoma haematobium*: development of novel microsatellite markers and their application to schistosomiasis control in Mali. Parasitology 138: 978–994. 21679489 10.1017/S0031182011000722

[9] Wright CA, Ross GC (1983) Enzymes in *Schistosoma haematobium* . Bull WHO 61: 307–316. 6222843 PMC2536131

[10] WHO Geneva, 1–45 WHO (2002) Prevention and control of schistosomiasis and soil-transmitted helminthiasis: report of a WHO expert committee. WHO Tech Rep Ser 912: 1–57. 12592987

[11] Fenwick A, Webster JP, Bosque-Oliva E, Blair L, Fleming FM, et al (2009) The Schistosomiasis Control Initiative (SCI): rationale, development and implementation from 2002–2008. Parasitology 136: 1719–1730. 19631008 10.1017/S0031182009990400

[12] Stothard JR, French MD, Khamis IS, Basanez MG, Rollinson D (2009) The epidemiology and control of urinary schistosomiasis and soil-transmitted helminthiasis in schoolchildren on Unguja Island, Zanzibar. Trans R Soc Trop Med Hyg 103: 1031–1044. 19409588 10.1016/j.trstmh.2009.03.024

[13] Savioli L, Gabrielli AF, Montresor A, Chitsulo L, Engels D (2009) Schistosomiasis control in Africa: 8 years after World Health Assembly Resolution 54.19. Parasitology 136: 1677–1681. 19765347 10.1017/S0031182009991181 PMC5642868

[14] Webster JP, Gower CM, Norton AJ (2008) Evolutionary concepts in predicting and evaluating the impact of mass-chemotherapy schistosomiasis control programmes on parasites and their hosts. Evol Apps 1: 66–83. 10.1111/j.1752-4571.2007.00012.x PMC3352399 25567492

[15] Norton AJ, Gower CM, Lamberton PHL, Webster BL, Lwambo NJS, et al (2010) Genetic consequences of mass human chemotherapy for *Schistosoma mansoni*: population structure pre- and post-praziquantel treatment in Tanzania. Am J Trop Med Hyg 83: 951–957. 20889898 10.4269/ajtmh.2010.10-0283 PMC2946775

[16] Rollinson D, Webster JP, Webster BL, Nyakaana S, Jørgensen A, et al (2009) Genetic diversity of schistosomes and snails: implications for control. Parasitology 136: 1801–11. 19631013 10.1017/S0031182009990412

[17] Morgan JAT, DeJong RJ, Kazibwe F, Mkoji GM, Loker ES, et al (2003) A newly-identified lineage of *Schistosoma* . Int J Parasitol 33: 977–985. 12906881 10.1016/s0020-7519(03)00132-2

[18] Gower CM, Shrivastava J, Lamberton PHL, Rollinson D, Webster BL, et al (2007) Development and application of an ethically and epidemiologically advantageous assay for the muti-locus microsatellite analysis of *Schistosoma mansoni* . Parasitology 134: 523–536. 17096873 10.1017/S0031182006001685 PMC2613677

[19] Le TH, Blair D, McManus DP (2000) Mitochondrial genomes of human helminths and their use as markers in population genetics and phylogeny. Acta Trop 77: 243–256. 11114386 10.1016/s0001-706x(00)00157-1

[20] Littlewood DTJ, Lockyer AE, Webster BL, Johnston DA, Le TH, et al (2006) The complete mitochondrial genomes of *Schistosoma haematobium* and *Schistosoma spindale* and the evolutionary history of mitochondrial genome changes among parasitic flatworms. Mol Phylogenet Evol 39: 452–467. 16464618 10.1016/j.ympev.2005.12.012

[21] Zarowiecki M, Huyse T, Littlewood DT (2007) Making the most of mitochondrial genomes - markers for phylogeny, molecular ecology and barcodes in *Schistosoma* (Platyhelminthes: Digenea). Int J Parasitol 37: 1401–1418. 17570370 10.1016/j.ijpara.2007.04.014

[22] Blair D, Le TH, Després L, McManus DP (1999) Mitochondrial genes of *Schistosoma mansoni* . Parasitology 119: 303–313. 10503256 10.1017/s0031182099004709

[23] Standley CJ, Kabatereine NB, Lange CN, Lwambo NJS, Stothard JR (2010) Molecular epidemiology and phylogeography of *Schistosoma mansoni* around Lake Victoria. Parasitology 137: 1937–49. 20561396 10.1017/S0031182010000788

[24] Morgan JA, Dejong RJ, Adeoye GO, Ansa ED, Barbosa CS, et al (2005) Origin and diversification of the human parasite *Schistosoma mansoni* . Mol Ecol 14: 3889–3902. 16202103 10.1111/j.1365-294X.2005.02709.x

[25] Webster BL, Culverwell CL, Khamis IS, Mohammed KA, Rollinson D, et al (2012) DNA barcoding of *Schistosoma haematobium* on Zanzibar reveals substantial genetic diversity and two major phylogenetic groups. Acta Trop http://dx.doi.org/10.1016/j.actatropica.2012.06.002. 10.1016/j.actatropica.2012.06.002 22721826

[26] Lockyer AE, Olson PD, Ostergaard P, Rollinson D, Johnston DA, et al (2003) The phylogeny of the Schistosomatidae based on three genes with emphasis on the interrelationships of Schistosoma Weinland, 1858. Parasitology 126: 203–224. 12666879 10.1017/s0031182002002792

[27] Tamura K, Peterson D, Peterson N, Stecher G, Nei M, Kumar S (2011) MEGA5: Molecular Evolutionary Genetics Analysis using maximum likelihood, evolutionary distance, and maximum parsimony methods. Mol Biol Evol 28: 2731–2739. 21546353 10.1093/molbev/msr121 PMC3203626

[28] Posada D (2008) jModelTest: Phylogenetic Model Averaging. Mol Biol Evol 25: 1253–1256. 18397919 10.1093/molbev/msn083

[29] Librado P, Rozas J (2009) DnaSP v5: A software for comprehensive analysis of DNA polymorphism data. Bioinformatics 25: 1451–1452. 19346325 10.1093/bioinformatics/btp187

[30] Kane RA, Rollinson D (1994) Repetitive sequences in the ribosomal DNA internal transcribed spacer of *Schistosoma haematobium*, *Schistosoma intercalatum* and *Schistosoma mattheii* . Mol Biochem Parasitol 63: 153–156. 8183315 10.1016/0166-6851(94)90018-3

[31] Webster BL (2009) Isolation and preservation of schistosome eggs and larvae in RNAlater® facilitates genetic profiling of individuals. Parasites and Vectors 2: 50. 19852777 10.1186/1756-3305-2-50 PMC2770516

[32] Gilbert E (2002) Coastal East Africa and the Western Indian Ocean: Long-Distance Trade, Empire, Migration, and Regional Unity, 1750–1970. The History Teacher 36.1. Available: http://www.historycooperative.org/journals/ht/36.1/gilbert.html. Accessed 2012 May 9.

[33] Webster BL, Southgate VR, Littlewood DTL (2006) A revision of the interrelationships of *Schistosoma* including the recently described *Schistosoma guineensis* . Int J Parasitol 36: 947–955. 16730013 10.1016/j.ijpara.2006.03.005

[34] Webster BL, Littlewood DTL (2012) Mitochondrial gene order in *Schistosoma* (Platyhelminthes: Digenea: Schistosomatidae). Int J Parasitol 42: 313–321. 23362512 10.1016/j.ijpara.2012.02.001

[35] Lawton SP, Hirai H, Ironside JE, Johnston DA, Rollinson D (2011) Genomes and geography: genomic insights into the evolution and phylogeography of the genus *Schistosoma* . Parasites and Vectors 4: 131. 21736723 10.1186/1756-3305-4-131 PMC3162923

[36] Kane RA, Stothard JR, Emery AM, Rollinson D (2008) Molecular characterization of freshwater snails in the genus *Bulinus*: a role for barcodes? Parasites and Vectors 1: 15. 18544153 10.1186/1756-3305-1-15 PMC2441610

[37] Karvonen A, Rellstab C, Louhi KR, Jokela J (2011) Synchronous attack is advantageous: mixed genotype infections lead to higher infection success in trematode parasites. Proc R Soc B 279: 171–176. 10.1098/rspb.2011.0879 PMC3223657 21632629

[38] Fallon PG, Doenhoff MJ (1994) Drug-resistant schistosomiasis: resistance to praziquantel and oxamniquine induced in *Schistosoma mansoni* in mice is drug specific. Am J Trop Med Hyg 51: 83–88. 8059919 10.4269/ajtmh.1994.51.83

[39] Ismail M, Botros S, Metwally A, William S, Farghally A, et al (1999) Resistance to praziquantel: direct evidence from *Schistosoma mansoni* isolated from Egyptian villagers. Am J Trop Med Hy 60: 932–935. 10.4269/ajtmh.1999.60.932 10403323

[40] Fenwick A, Webster JP (2006) Schistosomiasis: challenges for control, treatment and drug resistance. Curr Opin Infect Dis 19: 577–582. 17075334 10.1097/01.qco.0000247591.13671.6a

[41] Lamberton PHL, Hogan SC, Katbatereine NB, Fenwick A, Webster JP (2010) In vitro praziquantel test capable of detecting reduced in vivo efficacy in *Schistosoma mansoni* human infections. Am J Trop Med Hyg 83: 1340–1347. 21118946 10.4269/ajtmh.2010.10-0413 PMC2990056

[42] Feng Z, Curtis J, Minchella DJ (2001) The influence of drug treatment on the maintenance of schistosome genetic diversity. J Math Biol 43: 52–68. 12120867 10.1007/s002850100092

