# Correction: Genetic Diversity within *Schistosoma haematobium*: DNA Barcoding Reveals Two Distinct Groups

**DOI:** 10.1371/annotation/8b0ef43b-f45d-4098-8e6e-a92eebf50004

**Published:** 2013-02-26

**Authors:** Bonnie L. Webster, Aiden M. Emery, Joanne P. Webster, Anouk Gouvras, Amadou Garba, Oumar Diaw, Mohmoudane M. Seye, Louis Albert Tchuem Tchuente, Christopher Simoonga, Joseph Mwanga, Charles Lange, Curtis Kariuki, Khalfan A. Mohammed, J. Russell Stothard, David Rollinson

The cox1 accession number for Kenya on page 6 ("JQ397340") is incorrect. The correct accession number is "JQ397370." Additionally, this line is incorrectly printed twice.

The correct Supporting Information, Table 1 can be viewed here: 
