# Supplementary material for: Correction: Genetic Diversity within Schistosoma haematobium: DNA Barcoding Reveals Two Distinct Groups
Source: PLoS Negl Trop Dis. 2013 Feb 26;7(2):10.1371/annotation/8b0ef43b-f45d-4098-8e6e-a92eebf50004. doi: 10.1371/annotation/8b0ef43b-f45d-4098-8e6e-a92eebf50004 (PMC3586577; doi:10.1371/annotation/8b0ef43b-f45d-4098-8e6e-a92eebf50004)
Supplement: Supplementary file 1 [file pntd.8b0ef43b-f45d-4098-8e6e-a92eebf50004.s001.doc]

**Table S1 Sample and haplotype information**

| Country | **Locality** | **Locality Code*** | **Year of isolation / collection** | **Sample information** | **Haplotype Code**(H1)** | *cox*1 accession No. | No. individuals^ | *nad*1§ (Haplotype code**)(H1) | *nad1* accession No. | ITS |
| --- | --- | --- | --- | --- | --- | --- | --- | --- | --- | --- |
| Senegal | Guédé-Chantier | SE1 | 1983 | LPNHM -NHM127 (unknown) | SE1 (H1) | JQ397330 | 16 | SE1 (H1) | JQ595387 | Y |
|  | Mbodiene | SE2 | 1995 | LPNHM - NHM3290 (isolated from human urine samples) | SE2a (H1) | JQ397331 | 16 | SE2 | JQ595388 | Y |
|  |  | SE2 | 1997 | LPNHM -NHM3572 (isolated from naturally infected *B. globosus*) | SE2b | AJ519520”  JQ397332 | pooled worms |  |  | Y |
|  | Nder | SE3 | 2007 | 160 miracidia from 20 urine samples | SE3a (H1) | JQ397333 | 156 |  |  |  |
|  |  |  |  |  | SE3b | JQ595405 | 4 |  |  |  |
|  |  | SE3 | 2007 | 48 miracidia from 6 urine samples | SE3a (H1) | JQ397333 | 48 |  |  |  |
|  |  | SE3 | 2009 | 160 miracidia from 20 urine samples | SE3a (H1) | JQ397333 | 160 |  |  |  |
|  |  | SE3 | 2007 | 32 cercariae from 4 naturally infected snails | SE3a (H1) | JQ397333 | 32 |  |  |  |
|  | Temeye | SE4 | 2007 | 56 miracidia from 7 urine samples | SE4 (H1) | JQ397334 | 56 |  |  |  |
|  |  | SE4 | 2007 | 64 miracidia from 8 urine samples | SE4 (H1) | JQ397334 | 64 |  |  |  |
|  | Podor | SE5 | 2008 | 104 miracidia from 13 urine samples | SE5 (H1) | JQ397335 | 104 |  |  |  |
|  | Tambacounda | SE6 | 2009 | 96 miracidia from 12 urine samples | SE6a (H1) | JQ397336 | 96 |  |  |  |
|  |  | SE6 | 2009 | 96 miracidia from 12 urine samples | SE6a (H1) | JQ397336 | 96 |  |  |  |
|  |  | SE6 | 2009 | 96 miracidia from 12 urine samples | SE6a (H1) | JQ397336 | 94 |  |  |  |
|  |  |  |  |  | SE6b | JQ397337 | 2 |  |  |  |
|  | Kolda | SE7 | 2009 | 10 miracidia from 1 urine sample | SE7a (H1) | JQ397338 | 8 |  |  |  |
|  |  |  |  |  | SE7b | JQ397339 | 2 |  |  |  |
|  | Barkedji | SE8 | 2010 | 288 miracida from 12 urine samples | SE8a (H1) | JQ397340 | 280 |  |  |  |
|  |  |  |  |  | SE8b | JQ397341 | 4 |  |  |  |
|  |  |  |  |  | SE8c | JQ397342 | 4 |  |  |  |
|  | unknown | SE9 | 1985 | LPNHM - NHM571 (unknown) | SE9 (H1) | JQ397343 | 16 |  |  | Y |
| Mali | Niger Delta | MA1 | 1996 | LPNHM - NHM3375 (isolated from naturally infected *B. truncatus*) | MA1 | DQ157222” +  AJ271051”  JQ397344 | pooled worms | MA1 (H1) | DQ157222” |  |
|  | Niger Delta | MA2 | 1996 | LPNHM - NHM3390  (isolated from naturally infected *B. truncatus*) | MA2 (H1) | AY157209” | pooled worms |  |  |  |
|  |  | MA2 | 1996 | LPNHM - NHM3356  (isolated from naturally infected *B. truncatus*) | MA2 (H1) | JQ397345 | 16 | MA2 (H1) | JQ595389 | Y |
| Niger | Libore | NI1 | 2007 | 96 miracidia from 12 urine samples | NI1a (H1) | JQ397346 | 94 |  |  |  |
|  |  |  |  |  | NI1b | JQ397347 | 2 |  |  |  |
|  | Falmado | NI2 | 2007 | 40 miracidia from 5 urine samples | NI2 (H1) | JQ397348 | 40 |  |  |  |
| Gambia | unknown | GA1 | 1985 | LPNHM - NHM561  (unknown) | GA1 | JQ397349 | 16 | GA1 (H1) | JQ595390 | Y |
| Liberia | Wenshu | LB1 | 1987 | LPNHM - NHM1439 (isolated from naturally infected *B. truncatus*) | LB1 (H1) | JQ397350 | 16 | LB1 (H1) | JQ595391 | Y |
| Guinea Bissau | Gabu | GB1 | 1990 | LPNHM - NHM1895 (isolated from naturally infected *B. globosus*) | GB1 (H1) | JQ397351 | 16 | GB1 (H1) | JQ595392 | Y |
| Nigeria | Kano | NG1 | 1985 | LPNHM - NHM682 (isolated from naturally infected *B. truncatus*) | NG1 (H1) | JQ397352 | 16 | NG1 (H1) | JQ595393 | Y |
| Cameroon | Bessoum | CA1 | 2007 | LPNHM – no NHM no. (isolated from human urine samples) | CA1 (H1) | JQ397353 | 5 | CA1 (H1) | JQ595394 | Y |
|  |  |  |  | 192 miracida from 24 urine samples | CA1a (H1) | JQ397354 | 179 |  |  |  |
|  |  |  |  |  | CA1b | JQ397355 | 3 |  |  |  |
|  |  |  |  |  | CA1c | JQ397356 | 2 |  |  |  |
|  |  |  |  |  | CA1d | JQ397357 | 2 |  |  |  |
|  |  |  |  |  | CA1e | JQ397358 | 1 |  |  |  |
|  |  |  |  |  | CA1f | JQ397359 | 1 |  |  |  |
|  |  |  |  |  | CA1g | JQ397360 | 1 |  |  |  |
|  |  |  |  |  | CA1h | JQ397361 | 1 |  |  |  |
|  |  |  |  |  | CA1i | JQ397362 | 1 |  |  |  |
|  |  |  |  |  | CA1j | JQ397363 | 1 |  |  |  |
|  | Okuro | CA2 | 2007 | 10 miracidia from pooled urine samples | CA2 (H1) | JQ397364 | 10 |  |  |  |
|  | Loum | CA3 | 1990 | LPNHM - NHM1929 (isolated from naturally infected *B. truncatus*) | CA3 (H1) | JQ397365 | 16 |  |  |  |
|  |  | CA3 | 1990 | LPNHM - NHM1939  (isolated from naturally infected *B. truncatus*) | CA3 (H1) | JQ397365 | 16 |  |  |  |
|  | Barombi Mbo | CA4 | 1990 | LPNHM - NHM3640  (isolated from naturally infected *B. truncatus*) | CA4 (H1) | JQ397366 | 16 |  |  |  |
|  | Barombi Kotto | CA5 | 1990 | LPNHM - NHM1878  (isolated from naturally infected *B. truncatus*) | CA5 (H1) | JQ397367 | 16 |  |  |  |
| Egypt | ? | EG1 | 1988 | LPNHM - NHM1587 (unknown) | EG1 | JQ397368 | 16 | EG1 (H1) | JQ595395 | Y |
|  | Abbo Ramash | EG2” | ? | LP  (unknown) | EG2 | U22159” | pooled worms |  |  |  |
| Sudan | Gezira | SU1 | 1984 | LPNHM – NHM261 (unknown) | SU1 (H1) | JQ397369 | 16 | SU1 (H1) | JQ595396 | Y |
| Kenya | Taveta | KE2 | 2009 | 10 miracidia from pooled urines | KE2 (H1) | JQ397370 | 10 |  |  |  |
| Coastal Kenya | Rekeke | CK1 | 2007 | 39 miracidia from pooled urines | CK1a | JQ397371 | 20 |  |  |  |
|  |  |  |  |  | CK1b | JQ397372 | 8 |  |  |  |
|  |  |  |  |  | CK1c | JQ397373 | 4 |  |  |  |
|  |  |  |  |  | CK1d | JQ397374 | 2 |  |  |  |
|  |  |  |  |  | CK1e | JQ397375 | 2 |  |  |  |
|  |  |  |  |  | CK1f | JQ397376 | 1 |  |  |  |
|  |  |  |  |  | CK1g | JQ397377 | 1 |  |  |  |
|  |  |  |  |  | CK1h | JQ397378 | 1 |  |  |  |
|  | Kinango | CK2 | 2007 | 24 miracida from pooled urines | CK2a | JQ397379 | 15 |  |  |  |
|  |  |  |  |  | CK2b | JQ397380 | 5 |  |  |  |
|  |  |  |  |  | CK2c | JQ397381 | 2 |  |  |  |
|  |  |  |  |  | CK2d | JQ397382 | 2 |  |  |  |
|  | Nimbodze | CK3 | 2007 | 15 miracida from pooled urines | CK3a | JQ397383 | 5 |  |  |  |
|  |  |  |  |  | CK3b | JQ397384 | 5 |  |  |  |
|  |  |  |  |  | CK3c | JQ397385 | 4 |  |  |  |
|  |  |  |  |  | CK3d | JQ397386 | 1 |  |  |  |
| Tanzania | Mafia | Mafia | 2007 | LPNHM – no NHM no.  (Isolated from pooled urine samples) | Mafia1 | JQ082121 | 16 | Mafia1 | JQ595397 | Y |
|  |  |  |  |  | Mafia2 | JQ082122 | 4 | Mafia2 | JQ595398 |  |
|  | Mwanza | TA1 | 1998 | LPNHM - NHM4335 (unknown) | TA1a (H1) | JQ397387 | 16 | TA1 | JQ595402 | Y |
|  |  | TA1 | 2008 | 20 miracidia from pooled urines | TA1b | JQ397388 | 20 |  |  |  |
| Zanzibar* | Unguja | Zan | 2001 | LPNHM (isolated from urine samples) | Zan 1 | GU257334” | 56 | (Zan1) | GU257385” | Y |
|  |  |  |  |  | Zan 2 | GU257335” | 23 |  |  |  |
|  |  |  |  |  | Zan 3 | GU257336” | 22 |  |  |  |
|  |  |  |  |  | Zan 4 (H1) | GU257337” | 18 | Zan4 (H1) | GU257375” |  |
|  |  |  |  |  | Zan 5 | GU257338” | 13 |  |  |  |
|  |  |  |  |  | Zan 6 | GU257339” | 12 |  |  |  |
|  |  |  |  |  | Zan 7 | GU257340” | 9 |  |  |  |
|  |  |  |  |  | Zan 8 | GU257341” | 7 |  |  |  |
|  |  |  |  |  | Zan 9 | GU257342” | 7 |  |  |  |
|  |  |  |  |  | Zan 10 | GU257343” | 7 |  |  |  |
|  |  |  |  |  | Zan 11 | GU257344” | 6 |  |  |  |
|  |  |  |  |  | Zan 12 | GU257345” | 5 |  |  |  |
|  |  |  |  |  | Zan 13 | GU257346” | 3 |  |  |  |
|  |  |  |  |  | Zan 14 | GU257347” | 3 |  |  |  |
|  |  |  |  |  | Zan 15 | GU257348” | 3 |  |  |  |
|  |  |  |  |  | Zan 16 | GU257349” | 3 |  |  |  |
|  |  |  |  |  | Zan 17 | GU257350” | 3 |  |  |  |
|  |  |  |  |  | Zan 18 | GU257351” | 2 |  |  |  |
|  |  |  |  |  | Zan 19 | GU257352” | 2 |  |  |  |
|  |  |  |  |  | Zan 20 | GU257353” | 2 |  |  |  |
|  |  |  |  |  | Zan 21 | GU257354” | 2 |  |  |  |
|  |  |  |  |  | Zan 22 | GU257355” | 1 |  |  |  |
|  |  |  |  |  | Zan 23 | GU257356” | 1 |  |  |  |
|  |  |  |  |  | Zan 24 | GU257357” | 1 |  |  |  |
|  |  |  |  |  | Zan 25 | GU257358” | 1 |  |  |  |
|  |  |  |  |  | Zan 26 | GU257359” | 1 |  |  |  |
|  |  |  |  |  | Zan 27 | GU257360” | 1 |  |  |  |
| Malawi | Chembe | MW1 | 2005 | pooled cercariae from naturally infected *B. globosus* | MW1 (H1) | EU567129”  JQ397389 | pooled cercariae | MW1 (H1) | EU567138” |  |
|  |  | MW1 | 2005 | pooled cercariae from naturally infected *B. nyassanus* | MW1 (H1) | EU567130”  JQ397389 | pooled cercariae | MW1  (H1) | EU567139” |  |
|  | Likoma | MW2 | 2005 | pooled cercariae from naturally infected *B. globosus* | MW2a (H1) | EU567127”  JQ397390 | pooled cercariae | MW2  (H1) | EU567135” |  |
|  |  | MW2 | 2005 | pooled cercariae from naturally infected *B. globosus* | MW2b | EU567128”  JQ397391 | pooled cercariae | MW2 (H1) | EU567136” |  |
|  | ? | MW3 | 1998 | LPNHM - NHM880 (no information) | MW3 (H1) | JQ397392 | 16 | MW3 | JQ595399 | Y |
| Zambia | Simunjalala | ZA1 | 1991 | LPNHM -NHM2576 (isolated from urine samples) | ZA1 | JQ397393 | 16 | ZA1 |  | Y |
|  | Katunga | ZA2 | 2010 | 20 miracidia from 2 urine samples | ZA2 | JQ397394 | 20 |  |  |  |
|  | Kafue | ZA3 | 2008 | 10 cercariae from naturally infected *B. globosus* | ZA3 | JQ397395 | 10 |  |  |  |
|  | Kafue | ZA3 | 2008 | 8 miracidia from pooled urines | ZA3 | JQ397395 | 8 |  |  |  |
|  | Lisiko | ZA4 | 2008 | 32 miracidia from 4 urine samples | ZA4 | JQ397396 | 32 |  |  |  |
| South Africa | Durban | SA1 | 1986 | LPNHM - NHM812 (no information) | SA1 | JQ397397 | 16 | SA1 (H1) | JQ595401 | Y |
| Mauritius | Vallee Pitot | MU1 | 1992 | LPNHM –NHM2720 (isolated from urine samples) | MU1 | JQ397398 | 16 | MU1 | JQ595403 | Y |
| Madagascar | ? | MD1 | 1987 | LPNHM -NHM1864 (isolated from urine samples) | MD1 | JQ397399 | 16 | MD1 | JQ595404 | Y |

*Each locality is coded with a two-letter country code followed by a number

LPNHMSCAN = Laboratory passaged worms from SCAN: Schistosomiasis Collection at the Natural History Museum. NHM = The Natural History museum isolate number. LP = Laboratory passaged worms. Individual worms were not treated as individual samples but the different haplotypes found were incorporated into the analyses.

**Haplotypes are coded with their locality code and then a lowercase letter for each unique haplotype found within a locality. (H1) = the haplotype matches the most common *cox*1 haplotype

~ The haplotype is identical to the most common haplotype H1 (Y = yes),(N= no).

^This is the number of individual miracidia, cercariae or worms sampled from the same locality that had matching haplotypes

§The partial *nad*1 gene was amplified for these samples. (H1) = the haplotype matches the most common *nad*1 haplotype.

“Published data from Genbank (AJ519520, Kane et al., 2003; DQ157222, Littlewood et al., 2006; Le et al., 2000; AY157209, Lockyer et al., 2003; EU567127-EU567130 + EU567135-EU567139, Stauffer et al., 2008; GU257334-GU257360, GU257385, GU257375, Webster et al., in press; JQ082121-JQ082122 Stothard et al., in review).

ITS = The nuclear ITS1 + 2 was analysed from these samples.
